# Counteraction of Trehalose on N, N-Dimethylformamide-Induced *Candida rugosa* Lipase Denaturation: Spectroscopic Insight and Molecular Dynamic Simulation

**DOI:** 10.1371/journal.pone.0152275

**Published:** 2016-03-31

**Authors:** Xin Yang, Ling Jiang, Yigang Jia, Yi Hu, Qing Xu, Xian Xu, He Huang

**Affiliations:** 1 College of Biotechnology and Pharmaceutical Engineering, Nanjing Tech University, Nanjing 210009, PR China; 2 College of Food Science and Light Industry, Nanjing Tech University, Nanjing 210009, PR China; 3 College of Pharmaceutical Sciences, Nanjing Tech University, Nanjing 210009, PR China; University of Hyderabad, INDIA

## Abstract

*Candida rugosa* lipase (CRL) has been widely used as a biocatalyst for non-aqueous synthesis in biotechnological applications, which, however, often suffers significant loss of activity in organic solvent. Experimental results show that trehalose could actively counteract the organic-solvent-induced protein denaturation, while the molecular mechanisms still don’t unclear. Herein, CRL was used as a model enzyme to explore the effects of trehalose on the retention of enzymatic activity upon incubation in N,N-dimethylformamide (DMF). Results showed that both catalytic activity and conformation changes of CRL influenced by DMF solvent were inhibited by trehalose in a dose-dependent fashion. The simulations further indicated that the CRL protein unfolded in binary DMF solution, but retained the native state in the ternary DMF/trehalose system. Trehalose as the second osmolyte added into binary DMF solution decreased DMF-CRL hydrogen bonds efficiently, whereas increased the intermolecular hydrogen bondings between DMF and trehalose. Thus, the origin of its denaturing effects of DMF on protein is thought to be due to the preferential exclusion of trehalose as well as the intermolecular hydrogen bondings between trehalose and DMF. These findings suggest that trehalose protect the CRL protein from DMF-induced unfolding via both indirect and direct interactions.

## Introduction

In the past decade, biocatalysis has been established as a scalable and green technology alternative to traditional chemocatalysis in the production of industrial and specialty chemicals, foods as well as pharmaceuticals, both in the laboratory and on industrial scale [1]. Lipases (EC 3.1.1.3) have been proved to be versatile biocatalysts for catalyzing kinds of reactions, e.g., transesterification, esterification, asymmetric hydrolysis as well as organic synthetic reaction in water-restricted environment [2]. Natural lipases are often highly selective catalysts, but are not sufficiently tolerant to organic solvents. Polar solvents (e.g., N,N-dimethylformamide (DMF), dimethyl sulfoxide (DMSO), formamide) can easily take away water molecular from the protein surface and compete firmly for hydrogen bondings between proteins and water molecules, which often denature the protein state to an unfolded one with the loss of specific binding activity [3]. For example, an aqueous medium containing DMF even at levels of <10% would result in nearly 30% loss of enzyme activity [4]. From the industrial view points, how to improve the stability and the enzyme activity of lipases in typical organic solvent like DMF has became a prosperous and powerful area of research.

Various technical methods have been developed to enhance the performance of organic solvent tolerance of lipases from four main aspects, including directed evolution of lipase-producing strains, rational design of lipase genes, chemical modification and immobilization of existing lipase products [5,6]. For example, it is reported that *Proteus mirabilis* lipase has been successfully reengineered by directed evolution for improved thermostability as well as tolerance to DMF [7]. In our previous studies, the performance of lipases in organic solvents (e.g., DMF, methanol) has been improved by modifying with different kinds of functional ionic liquids as well as immobilizing onto the surface of mesoporous SBA-15 [8, 9]. It is also well-known that chemical chaperones as protein stabilizers can affect the stability and catalytic activity of different proteins [10]. Furthermore, these low-molecular-weight chemicals do not covalently modify the proteins. Such advantages would encourage the use of chemical chaperones to assist in the improvement of lipase activity in organic solvent.

Chemical chaperones are essentially osmolytes, which are usually made up of small organic molecules, such as methylamines (e.g., trimethylamine N-oxide [11] and betaine [12]), amino acids and derivatives [13], and polyols [14]. However, different chemical chaperones are known to interact with proteins in many different ways and the stabilizing effects also depend on the nature of the chemical chaperone used [15]. Among them, trehalose has been paid increasing attention because of its particularly efficient in protecting proteins from inactivation or denaturation caused by diverse stress conditions, such as desiccation, dehydration, heat, cold, and oxidation [16, 17]. Although the possible involvement of trehalose in protection against polar solvent such as DMF has never been studied, two findings led us to hypothesize that it may serve such a role: (i) large amounts of trehalose accumulate in microorganisms during the over-production of polar solvent (e.g., ethanol) [18, 19], and (ii) trehalose has a beneficial effect in the lyophilization process of protein [20]. However, the molecular mechanisms of the inhibiting action of trehalose on the lipase denaturation by polar solvent such as DMF is still not clear and remains a critical issue in the context of biotechnology.

*Candida rugosa* lipase (CRL) is a microbial lipase that constitutes the most important cluster of biocatalysts used in kinds of sectors, e.g., the pharmaceutical compounds, food products, surfactant, oleochemistry, and detergency industries [21]. Therefore, CRL has been used here as a model enzyme to inspect the influence of trehalose on the retention of enzyme activity upon incubation at different amounts of DMF. First, the influence of trehalose on catalytic activities as well as conformational changes of CRL induced by DMF was examined. And then, the thermodynamic behavior of the counteraction of trehalose on DMF-induced CRL denaturation was probed by analyzing the spectroscopic data. Finally, the molecular basis of DMF-induced CRL unfolding and the counteracting effects of trehalose on the unfolding were investigated in details based on molecular dynamic (MD) simulations. In view of all these analysis, discussions of the direct and indirect interactions have unveiled a molecular mechanism of trehalose-mediated stability of the CRL protein.

## Materials and Methods

### Materials

CRL (Type VII) was gained from Sigma-Aldrich (St. Louis, Missouri, USA) and stored at 0–4°C [22]. Trehalose, DMF, gum arabic, and olive-oil were available from Sinopharm Chemical Reagent Co., Ltd. All other chemicals were analytical-regent-grade.

### Enzyme and Mixed Solution Preparation

The CRL powder (1 g) was dissolved in 10 mL of the distilled water with magnetic stirring for 10 min at 4°C. Then the solution was centrifuged for the supernatant, and CRL solution was performed to remove excess salt by using a 10 kDa dialysis membrane. The sodium dodecyl sulfate-polyacrylamide gel electrophoresis (SDS-PAGE) was used to verify the purity of the enzyme (S1 Fig).

According to our preliminary experimental results based on the solubility of trehlaose at 40°C, the saturated concentration of trehalose to be mixed with DMF was 1.2 mol/L and thus the final concentration of trehalose was chosen between 0.5 and 1.2 mol/L in this study (data not shown). To minimize the experimental errors, the concentration of CRL was kept constant at 0.2 μmol/L with mixture of 0–40% (v/v) DMF solution.

### Lipase Activity Measurement

The activity of CRL was determined by olive oil hydrolysis [23]. A mixture of 8.5 g of gum arabic powder, 100 ml of deionized water, and 100 ml of olive oil was stirred overnight at room temperature to prepare the olive oil emulsion. The enzyme solution consisted of 10 ml phosphate buffer (0.025 mol/L, pH = 7.0), 10 ml olive oil emulsion and a moderate amount of enzyme. The reaction pH was maintained at 7.0, with the temperature strictly controlled at 40°C. The volume of NaOH consumed in 10 min was recorded, and then 10 mL acetone-ethanol was added to end the reaction. The hydrolysis activity of CRL was assayed by titration with 5 mM of NaOH using phenophthlein as indicator. One unit activity of lipase was defined to be the total amount of lipase required to release a titratable amount of fatty acid equivalent to 1 μmol NaOH per minute. All results represent means of three independent experiments (bars = standard deviation).

### Fluorescence Spectroscopy Studies

The fluorescence spectra of the CRL were monitored using a spectrofluorometer (Perkin-Elmer LS55, Shelton, CT, USA). CRL was added into the DMF solutions at different concentrations in the presence of trehalose (0, 0.5, 0.8, 1.0 mol/L). And emission spectra were registered in the emission ranges 320–450 nm with a 5 nm bandwidth after excitation at 280 nm. Then, a reference spectrum of the native CRL in distilled water was taken for comparison.

The evaluation of thermodynamic parameters was based on the equilibrium constant (*K*) for the two-state reversible transition as follows [24]:

$$N\overset{}{↔}D$$ (1)

where *N* and *D* represent the native state and the denatured one, respectively. The Δ*G*_*D*_ (Gibbs’s free energy change) for the two-state transition were calculated using Eq (2) in a similar way discussed before [25],

$$\Delta G_{D}=-RT\;\text{ln}\;K=-RT\;\text{ln}\;\left(\frac{F_{obs}-F_{N}}{F_{D}-F_{obs}}\right)$$ (2)

where *R* is the gas constant, *T* is the absolute temperature, *F*_*D*_ is the maximum fluorescence intensity at 335 nm of D state in 40% (v/v) DMF without trehalose, whereas *F*_*N*_ and *F*_*obs*_ are the physical parameters of fluorescence intensity at 335 nm of N state and any observed state, respectively.

### Molecular Dynamics Simulations

The initial structure for the simulations of CRL was taken from the Protein Data Bank (PDB code: 1LPO) with a resolution of 2.18 Å [26]. The topology files of DMF and trehalose were generated from the GlycoBioChem PRODRG2 server (http://davapc1.bioch.dundee.ac.uk/prodrg/) [27], with flexible simple point charge (SPC) water model. For simulating molecules, a force field, which includes the atomic charges, the charge group assignment defined based on GROMOS96.

The MD simulations were implemented through GROMACS 4.5.4 package at Hp Z800 workstation, using the GROMOS96 53a6 united atom protein force field [28]. The system used periodic cubic box that filled with 1.0 nm water molecule around and 17 positive ions (Na^+^) were added to neutralize charge [26]. Particle Mesh Ewald (PME) method was applied to copy the long-range electrostatic interactions with a cut-off of 10 Å. After 4,000 steps of energy minimization with steepest descent, the systems were then equilibrated with position restraints on heavy atoms of the protein for a minimum of 100 ps. The MD simulations were done to relax the protein and simulate unrestrictedly with a time step of 2 fs at 313 K and pressure of 1 atm. To assure that the counteraction of trehalose on the denaturing effect of DMF on protein, three different MD simulations of 10 ns were performed in initial systems (Table A in S1 File).

The normalized ratio of water oxygen atoms, *g*_NOW_, in different solvents was defined as follows:

$$g_{NOW}=\frac{n_{OW}\left(N_{OW}+N_{CD}+N_{OT}\right)}{\left(n_{OW}+n_{CD}+n_{OT}\right)N_{OW}},$$ (3)

where *n*_OW_: the numbers of water oxygen atoms, *n*_CD_: the numbers of DMF carbon atoms, and *n*_OT_: the numbers of hydroxyl oxygen atoms of trehalose, which are situated at the minimum distance from CRL, while the *N*_OW_: the numbers of water oxygen atoms, *N*_CD_: the numbers of DMF carbon atoms, *N*_OT_ the number of hydroxyl oxygen atoms of trehalose in the cubic box.

## Results

### Lipase activity measurements

**Fig 1:**
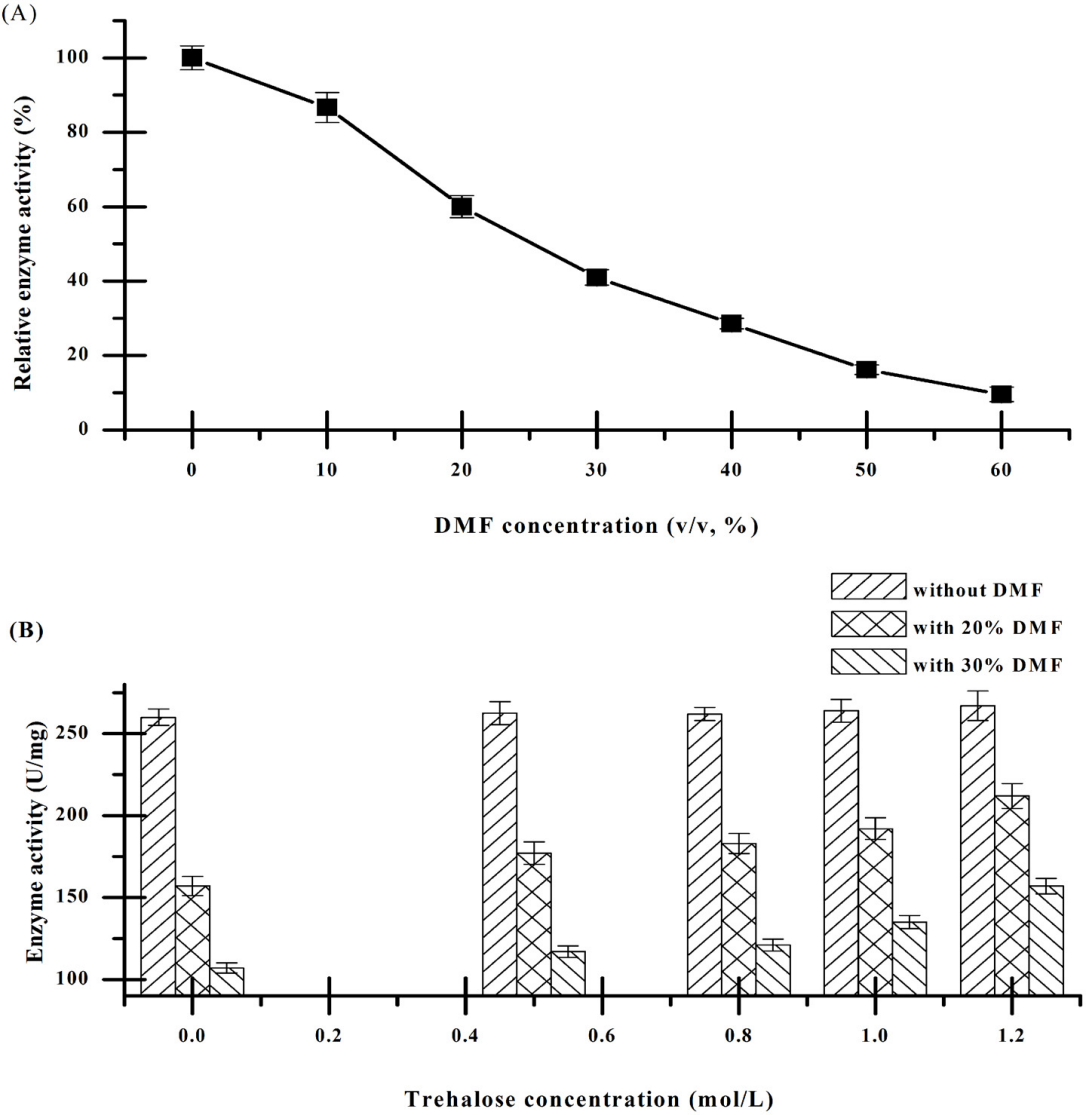
(A) Effects of different concentrations of DMF on the catalytic stabilities of CRL. (B) Effects of different concentrations of trehalose on the catalytic stabilities of CRL in 0, 20%, 30% (v/v) DMF.

As shown in Fig 1A, the specific activities of CRL were decreased almost linearly with the concentration of DMF increasing. The residual activity of the control (without DMF) was defined as 100%. Within 20% and 30% DMF, the CRL retained nearly only half of the original activity. Fig 1B showed that the addition of trehalose provided stabilizing effects, which remarkably increased the catalytic stability of CRL in DMF. When the concentration of trehalose reached upto 1.2 mol/L, the activity of CRL in 20% DMF retained 80% of the original activity, which was 35% higher than that in the absence of trehalose.

### Inhibitory effects of trehalose on conformational changes of CRL induced by DMF

**Fig 2:**
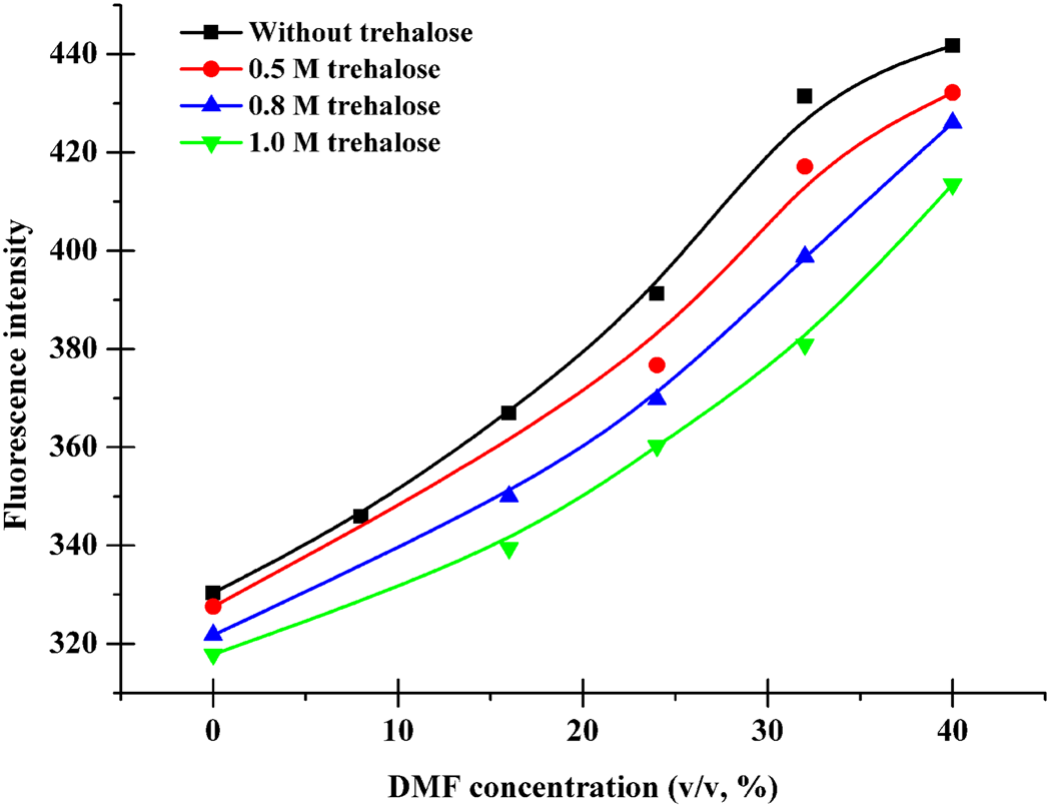
Fluorescence emission changes of CRL. Plots of fluorescence intensity at an emission wavelength of 335 nm and an excitation wavelength of 280 nm versus DMF concentration at different trehalose concentrations.

In order to fully understand the molecular mechanism of the protective action of trehalose, changes in the intrinsic fluorescence spectra of CRL preparation in DMF with addition of trehalose were determined. As there are four tryptophans (i.e., Trp134, Trp202, Trp236, Trp504) in the sequence of CRL and the intrinsic tryptophan fluorescence is considered to character conformational changes of the corresponding protein [29], the Trp residues fluorescence of CRL is therefore monitored by fluorescence spectroscopy to represent its conformational changes induced by DMF. At different DMF solutions, an emission spectrum with a peak around 335–340 nm was probed (S2 Fig). Nevertheless, when trehalose was added into the DMF solutions at the corresponding different concentrations, there was a litter blue shift of the peak (S3 and S4 Figs). Furthermore, DMF-induced denaturation of CRL with and without trehalose was followed by measuring changes in the fluorescence intensity at an emission wavelength 335 nm with excitation at 280 nm, as a function of DMF concentration (Fig 2). As can be seen in Fig 2, the fluorescence intensity increased rapidly along with the DMF concentration increasing, indicating that Trp residues are more exposed to the organic solvent. However, it can also be found in Fig 2 that the peak fluorescence intensity of DMF-denatured CRL decreases remarkably with increasing trehalose concentration from 0.5 mol/L to 1.0 mol/L. It suggests that higher concentration of trehalose hinders the exposure of Trp residues to solvent caused by DMF more effectively.

### Thermodynamic analysis of counteraction of trehalose on DMF-induced CRL denaturation

**Fig 3:**
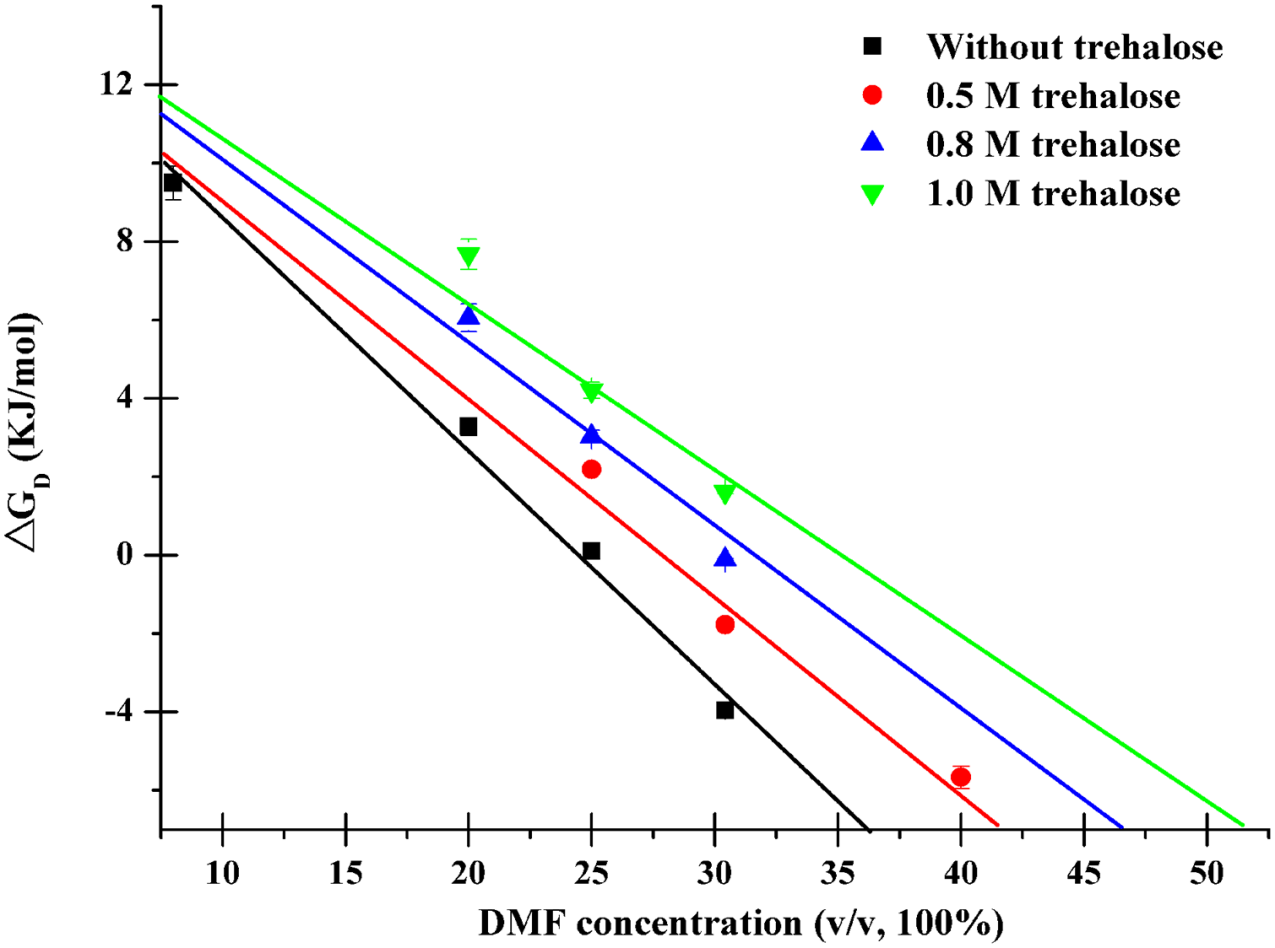
Plots of ΔG_D_ as a function of DMF concentration at different trehalose concentrations.

Fig 3 showed the plot of Δ*G*_*D*_ against the DMF concentration at different trehalose concentrations with a linear regression line fitted to the data. As can be seen in Fig 3, there was a linear decrease in the free energy change for DMF-induced protein denaturation with increasing DMF concentration in both absence and presence of trehalose (0.5, 0.8 and 1.0 mol/L). It implys that DMF lowers the free-energy barrier heights for the transition from the native state to denatured one, which is in proportion to the increased concentration. Thus, the native CRL protein is easier to be denatured to the unfolded state with the DMF concentration increased, which means that the CRL extends more extended at higher DMF concentration. However, in the presence of 0.5 mol/L trehalose, the values of Δ*G*_*D*_ are slightly larger than that at the corresponding DMF concentrations without trehalose. Furthermore, the Δ*G*_*D*_ values increase more evidently than that at the corresponding DMF concentrations without trehalose when increasing the trehalose upto 0.8 and 1.0 mol/L, respectively. It can be speculated that trehalose hinders the transition from the native to denatured sates induced by DMF more effectively at higher concentrations.

### Molecular dynamic simulation of CRL conformational properties in different solvent environments

**Fig 4:**
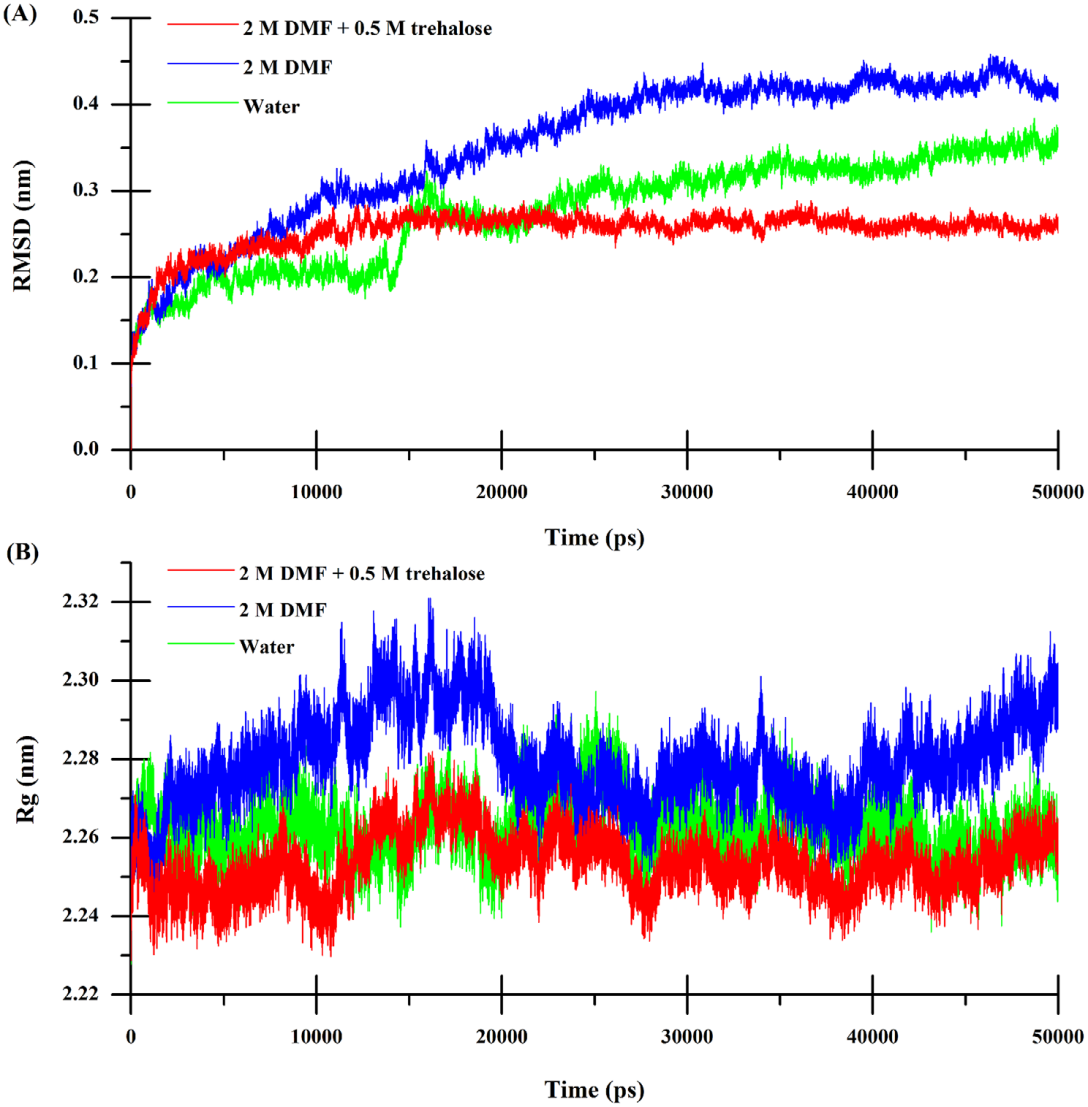
(A) RMSD from the initial native structure for C*α* atoms and (B) Rg as a function of time for the simulations in different solvent environments.

In order to quantitatively describe the capacity of trehalose-mediated protein stability, two structure parameters, the RMSD from the initial native state of C*α* atoms and the radius gyration (Rg), were applied to on behalf of the conformational changes of the CRL protein in different solvent environments. The C*α*-RMSD and Rg were calculated and displayed in pure water, the binary DMF solution, and the ternary solution mixed with DMF and trehalose, respectively, on the basis of time in Fig 4A and 4B. It is shown from Fig 4A that the values of C*α*-RMSD reached equilibrium after 25 ns in water, indicating that CRL maintains its native conformation. On the other hand, C*α*-RMSD values in binary DMF solution increased upto 3 Å after 15 ns, however, with the addition of trehalose, C*α*-RMSD values maintained around 2.5 Å in binary DMF solution all the time. It is speculated that DMF induces the structure conformation of CRL changed, which is in accordance with our previous experimental results. However, the values of RMSD in the ternary solution were most similar to those in pure water (Fig 4A). The above difference is even more obvious when considering the Rg values of CRL, as showed in Fig 4B. It is apparent that the Rg values in the ternary solution and water are very much comparable whereas the same in binary DMF solution is much higher from the beginning of the simulation, which is confirmed that trehalose has a super ability to counteract the DMF-induced protein denaturation.

**Fig 5:**
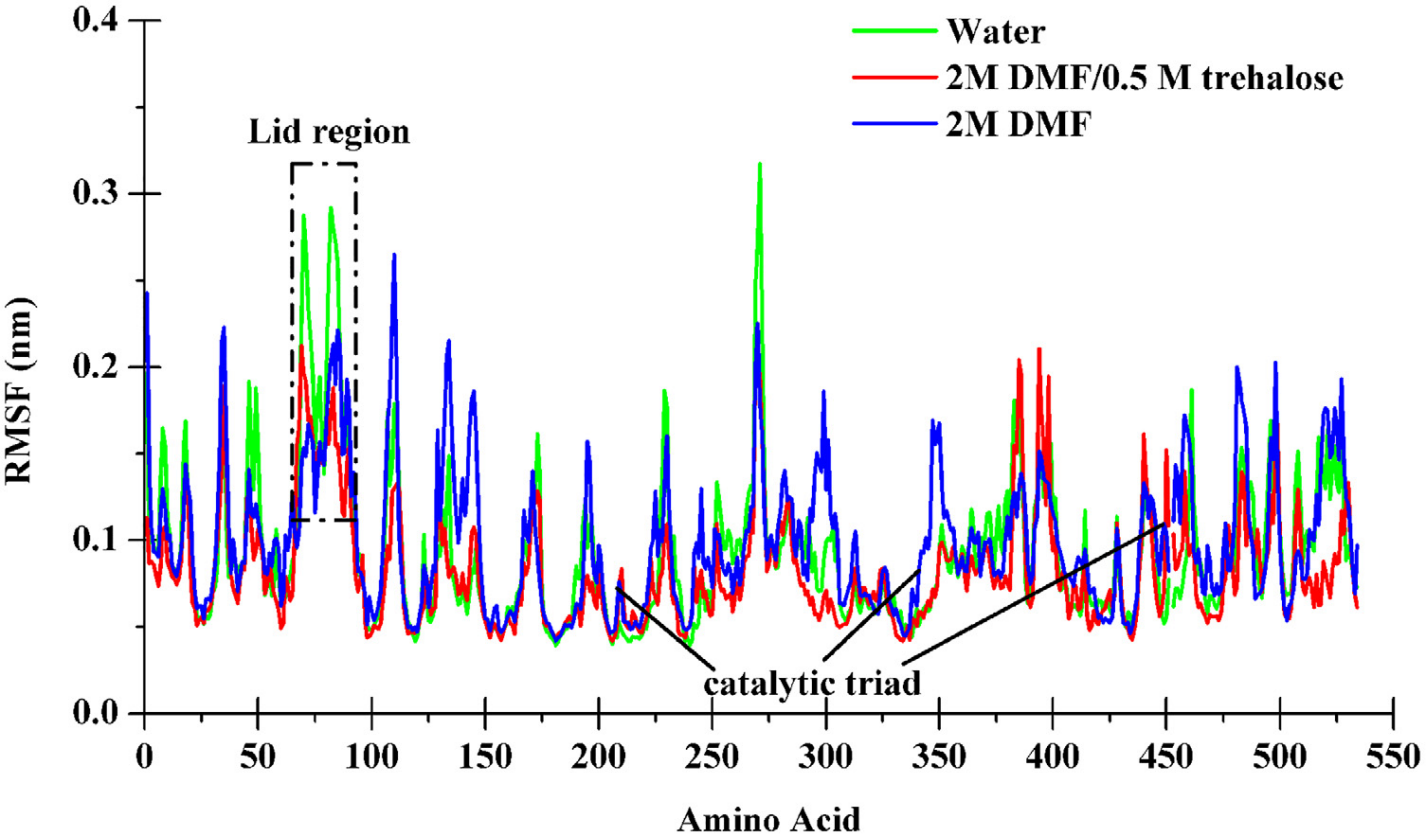
RMSF in simulations of CRL. The data were indicated the flexibility of CRL calculated from the last 6 ns of simulations in pure water (black), in binary DMF solution (blue), and in ternary DMF/trehalose solution (red).

To further evaluate the flexibility of CRL in different solvents, the root mean squared fluctuate (RMSF) per residue were estimated from the simulations. In binary DMF system, the overall flexibility was significantly higher than in the water, while the addition of trehalose decreased the overall flexibility of CRL (Table 1). In above three systems, CRL showed higher flexibility of surface residues and lower flexibility in the core and the active site (Fig 5). As can be seen in Fig 5, the region of lid was a major unstable surface element of CRL, which forms the entrance to the active sites (the catalytic triad residue Ser209, His449, Glu341) and involves a distorted helical turn (residue 69 to residue 72) as well as an α-helix (residue 75 to residue 84). In binary DMF solution, CRL showed lower flexibility of lid region, which, however, tended to significantly increase with the addition of trehalose. Contrary to this, the flexibility of the active sites slightly increased within DMF, while decreased when adding trehalose. In fact, more rigid in the catalytic triad region will be less able to adapt to the substrates entering the active site pocket, which could be improved to some extent with the addition of trehalose.

**Table 1:** Total RMSF (sum of RMSF per residue) of CRL averaged over the last 6 ns of each simulation in three solvents.

CRL in solvent	Total RMSF (nm)
**Water**	**81.40**
**Water+DMF**	**86.88**
**Water+DMF+Trehalose**	**59.96**

### Molecular dynamic simulation of preferential interaction in different solvent environments

**Fig 6:**
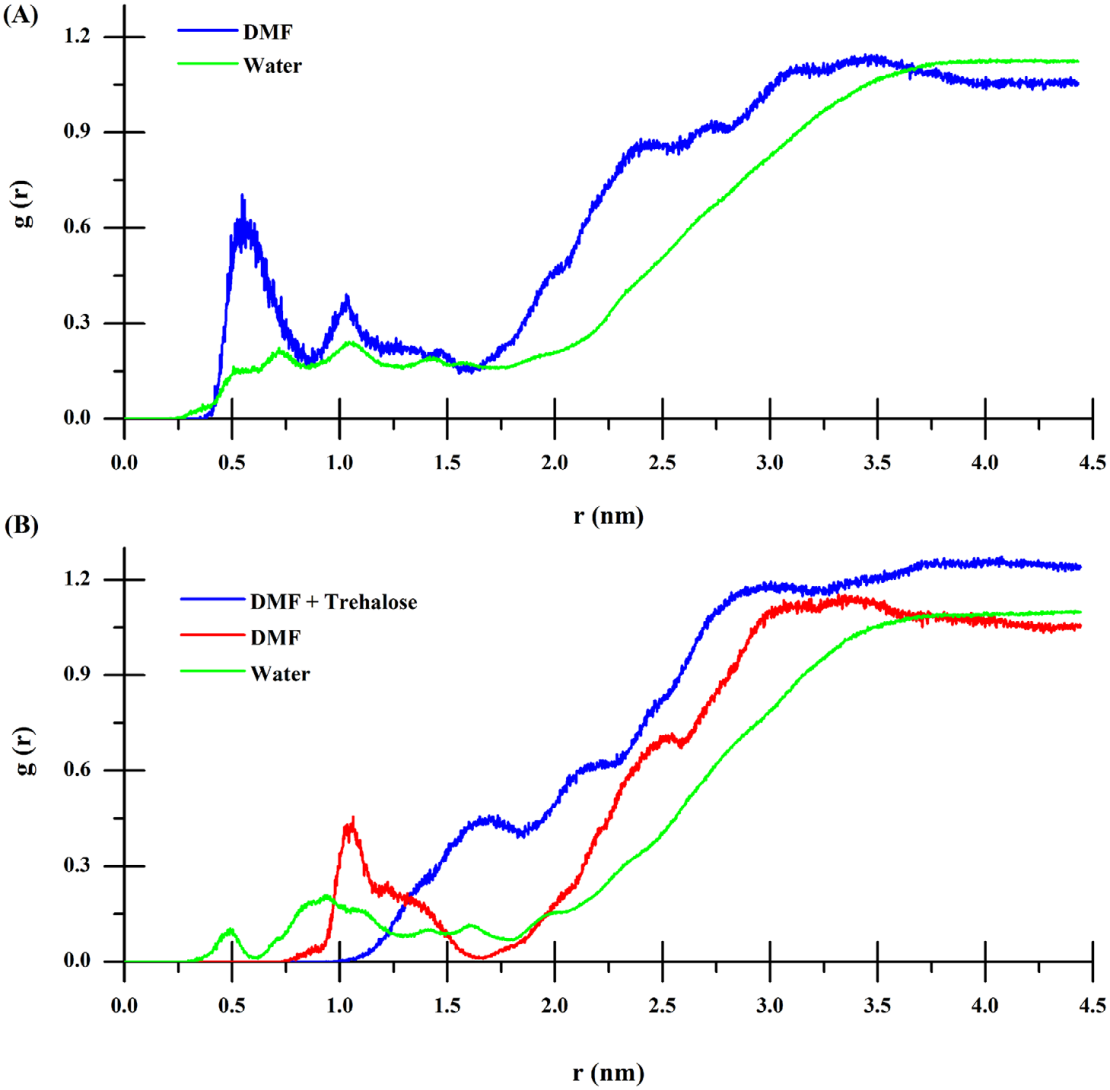
Radial distribution functions. RDFs for water oxygen, DMF carbon, and trehalose hydroxyl oxygen around the center of mass (COM) of the protein as a function of the distance *r* from COM of the protein in binary DMF solution (A) and a ternary DMF/trehalose solution (B) over the last 6 ns of the simulation.

To study the preferential interactions of salvation of CRL in different solutions, the radial distribution functions (RDFs) between the center of mass (COM) of CRL and different atoms (water oxygen, DMF carbon and trehalose hydroxyl oxygen) are computed. RDFs on the basis of the distance r from the COM of CRL were calculated in a MD simulation at the same thermodynamic conditions. Fig 6A showed that in binary DMF solution, the DMF density from the COM of CRL was more than that of water in the approximate range 0.4‒3.5 nm, indicating that numerous DMF molecules distribute about the CRL protein and expel water from the CRL surface. It could be speculated that DMF can directly interact with CRL, which may play a role of inducing the denaturation of CRL. However, with the additive of 0.5 mol/L trehalose, water density was increased in the 9 Å range from the COM of CRL (Fig 6B). Meanwhile, the DMF density decreased significantly compared with the corresponding values in binary DMF solution. In other words, DMF molecules were expelled from the CRL surface, and a spot of water molecules existed in the 5 Å range from the COM of CRL. Furthermore, trehalose just distributed in the range of more than 10 Å from the COM of CRL. This means that trehalose was preferentially excluded from the domain of the CRL protein, which was proved as the primary cause of stabilizing the protein by trehalose. Hydrogen bonding interaction among different solutions has also an important role in protein stabilization. Hence, the different types of mean hydrogen bond numbers were calculated and summarized in Table 2 to examine the protein salvation closely. It shows that with DMF in the solution, the hydrogen-bond numbers in the second structure (e.g., the helix of the peptide) of CRL was 380, while in the pure water system the corresponding value was 400, which indicates the helical structure of CRL was lost in binary DMF solution. Interestingly, the number of intramolecular hydrogen bonds in the second structure of CRL was as much as that of the pure water system in the ternary DMF and trehalose solution. The result indicated that trehalose had the ability to stabilize protein and counteract DMF-induced denaturation. The exclusion of DMF from CRL surface could also be proved with the fact that the CRL-DMF hydrogen bond number was decreased from 749 to 48 in the presence of trehalose. We noted that only 6 hydrogen bonds were formed between trehalose and CRL protein, which was much fewer than those could form with the protein. Furthermore, it could be found that a great number of DMF-trehalose hydrogen bonds formed in ternary solution (about 4), which might be a reason of the reduction of the CRL-DMF hydrogen bonds discussed above.

**Table 2:** Mean number of hydrogen bonds between different molecules in different solutions ^a^.

System	CRL-CRL	CRL-DMF	CRL-Tre	DMF-Tre
**Water**	**400 ± 12**	**-**	**-**	**-**
**Water+DMF**	**380 ± 18**	**749 ± 24**	**-**	**-**
**Water+DMF+Tre**	**411 ± 12**	**48 ± 8**	**6 ± 2**	**4 ± 2**

^a^ All values were averaged over the whole MD simulations.

**Fig 7:**
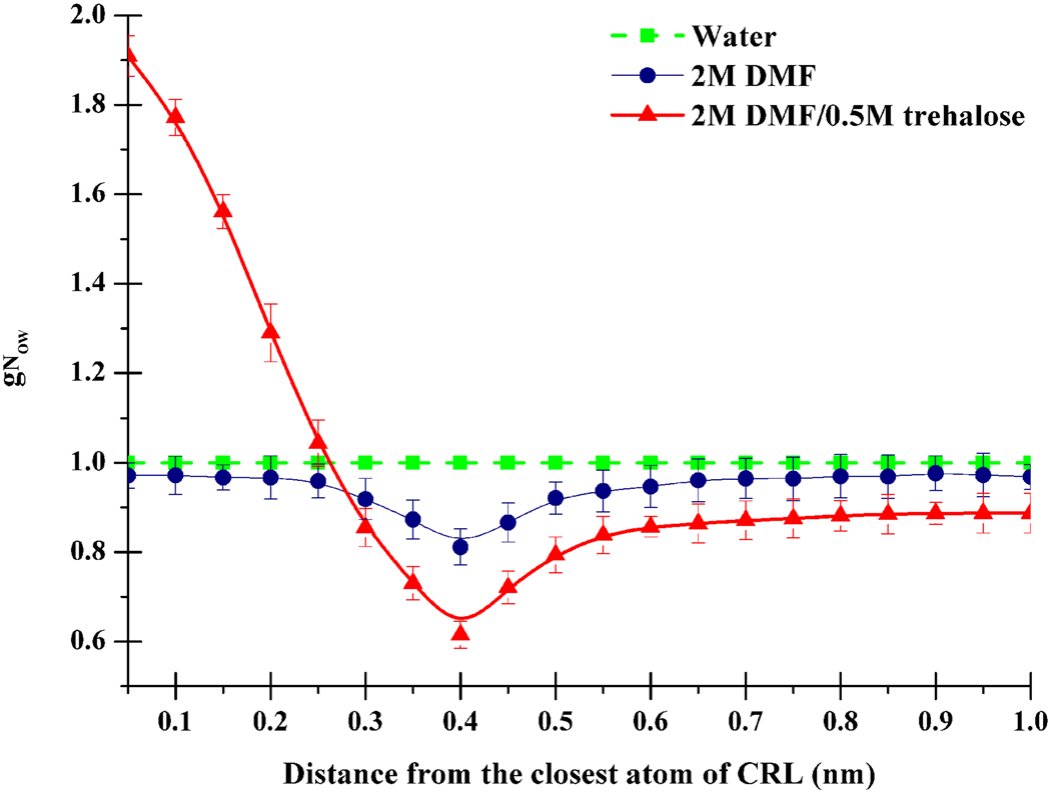
Preferential exclusion properties of water. In different solutions, time-averaged normalized ratio of water oxygen atoms, *g*N_OW_, is presented as a function of the minimal distance to any atoms of CRL.

To further study the preferential hydration of CRL in different solutions, the relative distribution of water molecules from MD trajectories around CRL protein was characterized. We calculated the time-meaned normalized ratio of water oxygen atoms *g*_NOW_ [Eq (3)] in a similar manner with Lerbret et al [30]. The intervals were chosen in 0.5 Å steps between 0.5 and 10.0 Å from the closest atoms of the protein. As can be seen in Fig 7, *g*_NOW_ was higher than 1 in a distance of around 2.5 Å from the closest atoms of CRL in the ternary DMF/trehalose solution, whereas lower than 1 throughout the distances examined in binary DMF solution. It is proved that DMF molecules are excluded from the CRL surface by trehalose and the protein is preferentially hydrated.

## Discussion

The technological utility of lipases can be strengthened significantly by using them in organic solvents rather than the natural aqueous media [31]. In previous works, it have already revealed that in this seemingly harsh environments lipases can reduce the side reactions, control the substrate specificity, catalyze reactions impossible in aqueous solutions, and even show novel behavior such as 'molecular memory' [32]. However, the catalytic activity of lipases in organic systems is usually orders of magnitude lower than in aqueous solutions. Numerous studies over the last two decades have contributed to the explanation of the mechanisms for this difference in activity [33].

Chemical chaperones as osmolytes added to aqueous media of biomolecules are well-known to affect protein stability and biochemical equilibria [34]. Both *in vivo* and *in vitro* experiments have demonstrated that trehalose can act as a protective agent of proteins against thermal inactivation and chemical denaturation, while the underlying mechanisms that makes trehalose an effective bioprotectant is still elusive [35, 36]. It is obviously that the researches of trehalose to offset the DMF-induced protein denaturation are extremely limited. Herein, we systematically studied the catalytic stabilities of CRL in DMF and thermodynamic behaviors of CRL against DMF denaturation by trehalose based on the spectroscopic insight. The biological activity measurement carried out here verified the inhibitory effect of trehalose against protein conformational changes with the loss of specific activity caused by DMF was a dose-dependent phenomenon. Furthermore, a thermodynamic analysis of the offset of trehalose on DMF-induced CRL denaturation was performed at different DMF concentrations with and without trehalose. From the fluorescence measurements, we found that the barrier heights of free-energy for the transition from the native to denatured state would be enhanced by increasing the trehalose concentration. That is, trehalose hinders the transition from the native to denatured sates induced by DMF more effectively at higher concentrations. Therefore, the CRL protein becomes more stable at higher trehalose concentrations. The thermodynamic mechanism for the inhibition of DMF-induced protein unfolding by trehalose in the observable phases is similar to that for the acid-induced protein unfolding inhibited by trehalose [37]. Besides, Pazhang et al. also found that the stabilization of thermolysin at various DMF concentrations by trehalose was mainly due to a decrease in the unfolding rate constant [38]. Moreover, the protein unfolding depended on the solution viscosity, and it could be inhibited by solution viscosity [39, 40]. It is known that the addition of trehalose increases the solution viscosity with the trehalose concentration increased [41]. Therefore, it can be speculated that the increase in the solution viscosity with increasing trehalose concentration could also partly result in the decrease in the DMF-induced unfolding rates of CRL, which however is not a critial parameter for the protection of protein against DMF denaturation by trehalose.

From the perspective of molecular level, there are roughly two classes of interaction models being put forward to interpret the peculiarity of trehalose: direct and indirect interactions [36]. Most versions of the direct interaction model posit that trehalose binds to protein via hydrogen bonds and stabilizes the native state. In the indirect interaction model, however, it is proposed that trehalose acts indirectly by enhancing the hydrophobic interactions between pairs of hydrophobic groups and stabilizing the native state. Preferential exclusion is one of the three common hypotheses recognized as the mechanism for the stabilization of protein by trehalose [36]. The mechanism implies that there is an increase of water molecules in the hydration shell around the CRL protein since trehalose molecules are expulsed from the surface of CRL, The above intriguing discovery of the ability of trehalose to stabilize proteins and offset DMF-induced denaturation further hinted our MD simulations of the system to provide more detailed structural information. The overall topology was preserved in the presence of trehalose in contrast to DMF simulations (Figs 4 and 5). Our results showed that both the Cα-RMSD and Rg values for CRL in the presence of 0.5 mol/L trehalose in 2 mol/L DMF were most similar to CRL in pure water (Fig 4). However, the RMSD of CRL in binary DMF solution fluctuated after 15 ns, while the Rg value was in a steady uptrend even from the very beginning. Fluctuations in the backbone occur chiefly in regions that are usually susceptible to DMF, for example, the active-sites and the lid region (Fig 5), which are perturbed markedly with addition of trehlaose. It is in consistent with recent report referring to the inhibition effect of acid-induced protein denaturation by trehalose [37], but it reveals that trehalose does not act by counteracting urea-water interactions in model protein chymotrypsin inhibitor 2 [42]. However, trehalose did indeed disrupt protein-DMF hydrogen bonds (Table 2). The averaged energies of hydrogen bonds between the protein and DMF increased greatly from less than 21 kJ/mol to over 27 kJ/mol with the addition of trehalose (Table B in S1 File), which is similar to the previous experimental results referring to other polyols [14]. The lower number of intramolecular contacts as well as the increased flexibility on account of trehalose can be considered an important factor in determining the recovery of CRL activity in DMF solution [43]. In our DMF-denaturation simulation, water molecules firstly solvated the hydrophobic COM of CRL. The DMF followed when the COM opened sufficiently. It means that water was the first denaturant. Trehalose seems to prevent the initial water attack by means of occluding DMF from CRL surface, then inducing thermodynamic stabilization (Fig 6). Furthermore, the preferential hydration of CRL in the presence of trehalose can be found by analyzing the distribution of water molecules around CRL (Fig 7). It is indicated that the protein is preferentially hydrated (in other words, DMF is expelled from the protein), with *g*_NOW_ greater than 1 in a distance of 0.25 nm from the closet atoms of the protein. Therefore, the preferential exclusion of trehalose is the origin of its counteraction effects on the DMF-induced CRL denaturation.

It is known that the information of peptide in folded, unfolded state could be obtained from the hydrogen bonds within the protein. Trehalose is a compound of polyol including 8 different hydroxyl groups, which may directly interact with protein through hydrogen bonds. In the meantime, the methyl groups of DMF could provide hydrogen atoms and its aldehyde group could accept hydrogen atoms by forming two and/or more intermolecular hydrogen bonds [44]. It indicates that trehalose can form hydrogen bonds with the methyl or aldehyde groups of DMF through its hydroxyl groups. Indeed, as much as 4 hydrogen bonds could be formed in the ternary DMF/trehalose solution (Table 2). That is, trehalose has an ability to bind DMF through hydrogen-bond, therefore DMF molecules can also be excluded from the surface of the protein together with the preferential exclusion of trehalose in the ternary DMF/trehalose system. This phenomenon is different from the earlier reported conclusions where it is declared that the preferential exclusion of trehalose is the only reason for stabilizing protein [45]. The presence of a significantly large amount of hydrogen bonds between trehalose and DMF directs us to propose that DMF molecules would be reduced in CRL solvation shell because of the preferential solvation of trehalose molecules, which prevent to denature CRL protein [46]. It is similar to the behavior of TMAO, in which the urea concentration at the protein surface is reduced because of the increasing hydrogen-bond between urea and TMAO, thus offseting the deleterious effect of urea on protein conformation. Thus, we could conclude that the supplement of trehalose results in the expelling of urea from the protein surface because of the direct interactions between trehalose and DMF.

To conclude, both experimental and all-atom MD simulation studies were conducted to research the offsetting effects of trehalose on DMF-induced denaturation of CRL protein. It is confirmed that the activity of CRL decrease by DMF is inhibited by trehalose in a dose-dependent manner. We also found that the free energy changes for DMF-induced CRL protein denaturation increase with increasing trehalose concentration from the fluorescence measurements, that is, the protein becomes more stable at higher trehalose concentrations in different DMF concentrations. Through the Cα-RMSD and Rg calculation, we found that CRL unfolded absolutely in binary DMF solution. By addition of 0.5 mol/L trehalose, the native state of CRL is well kept. In binary DMF solution, DMF molecules compete the protein-bound water molecules at the protein surface and denature the protein directly. In contrast, in the ternary DMF/trehalose system, trehalose interacted with DMF by hydrogen bonds directly, then DMF molecules are expelled from CRL surface because of the preferential exclusion of trehalose, resulting in the preferential hydration of CRL. Therefore, the above study allows us to infer that trehalose offset the influence of DMF-induced protein denaturation with two important factors: (i) preferential exclusion of trehalose and (ii) direct interaction between trehalose and DMF by hydrogen-bond interactions. In addition, the formation of numerous DMF-trehalose hydrogen bonds in ternary solution further supports the preferential salvation model, which points out that in the offset of DMF-induced protein denaturation by osmolyte, such as trealose here, it is the preferential solvation of the osmolyte that prevents DMF molecules to attack the protein indirectly.

## Supporting Information

S1 Fig SDS-PAGE analysis of purified CRL. Aliquots of the protein samples were analyzed on a 12.5% polyacrylamide gel under denaturing conditions. Lane 1 purified CRL using 10 KDa dialysis membranes to remove the excess salts.
(TIF)

S2 Fig Fluorescence spectra of CRL as a function of DMF concentration in the absence of trehalose. CRL was dissolved in 20% (v/v), 30% (v/v) DMF with addition of 0.5 M and 1.0 M trehalose, respectively. The fluorescence spectrum of CRL in water was as the control. The final CRL concentration was 5 μM.**(c)**
(TIF)

S3 Fig Fluorescence spectra of CRL as a function of DMF concentration in the presence of 0.50 M trehalose. CRL was dissolved in 20% (v/v), 30% (v/v) DMF with addition of 0.5 M and 1.0 M trehalose, respectively. The fluorescence spectrum of CRL in water was as the control. The final CRL concentration was 5 μM.**(c)**
(TIF)

S4 Fig Fluorescence spectra of CRL as a function of DMF concentration in the presence of 1.0 M (C) trehalose. CRL was dissolved in 20% (v/v), 30% (v/v) DMF with addition of 0.5 M and 1.0 M trehalose, respectively. The fluorescence spectrum of CRL in water was as the control. The final CRL concentration was 5 μM.**(c)**
(TIF)

S1 File Table A. Summary of Simulation Systems. Table B. Energies of hydrogen bonds (*E*_hb_) of CRL in binary and ternary solutions.
(DOCX)
