# Supplementary figures and images for: Counteraction of Trehalose on N, N-Dimethylformamide-Induced Candida rugosa Lipase Denaturation: Spectroscopic Insight and Molecular Dynamic Simulation
Source: PLoS One. 2016 Mar 31;11(3):e0152275. doi: 10.1371/journal.pone.0152275 (PMC4816565; doi:10.1371/journal.pone.0152275)

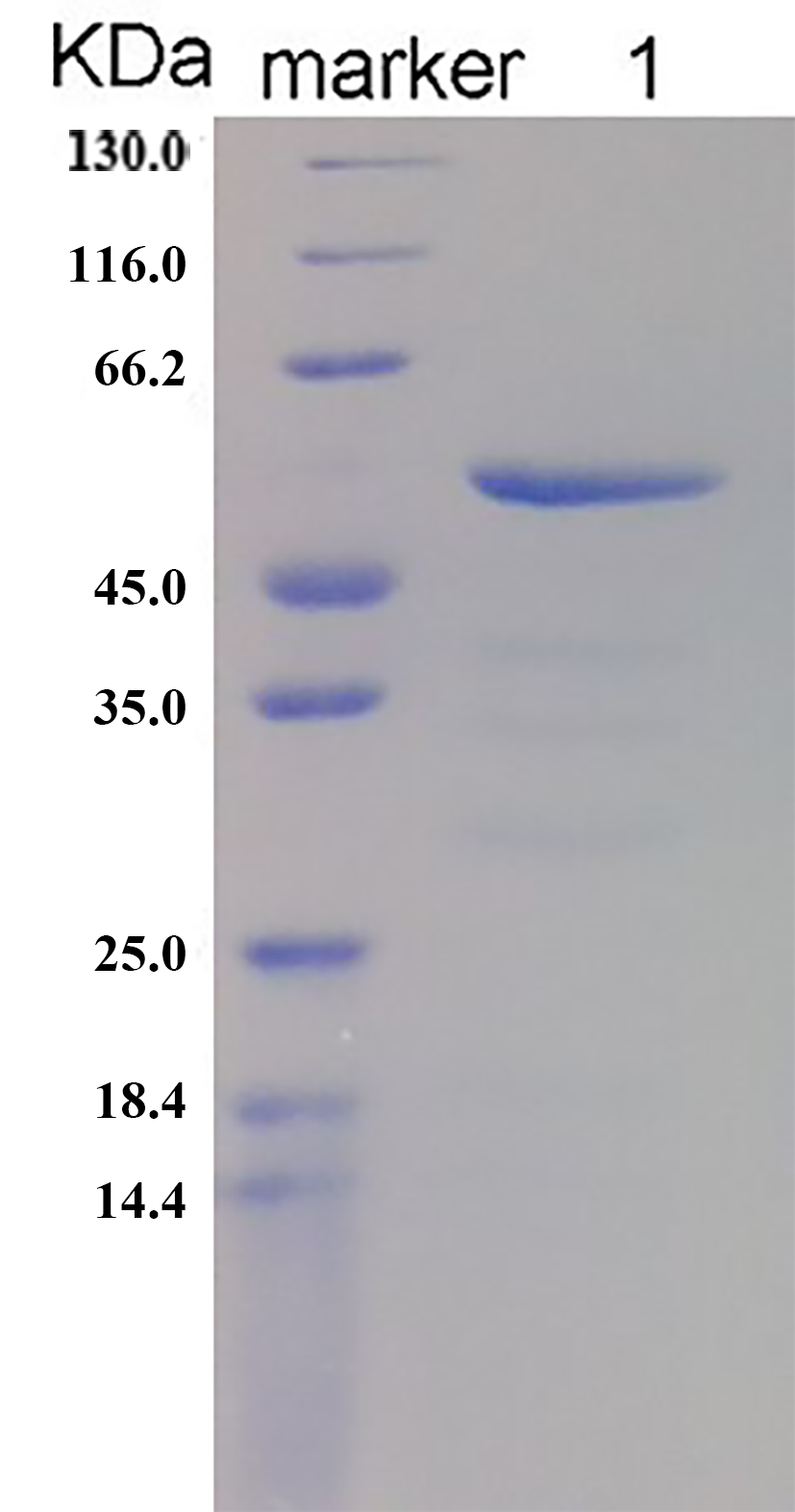

Supplement: S1 Fig — Aliquots of the protein samples were analyzed on a 12.5% polyacrylamide gel under denaturing conditions. Lane 1 purified CRL using 10 KDa dialysis membranes to remove the excess salts. (TIF) [file pone.0152275.s001.tif]

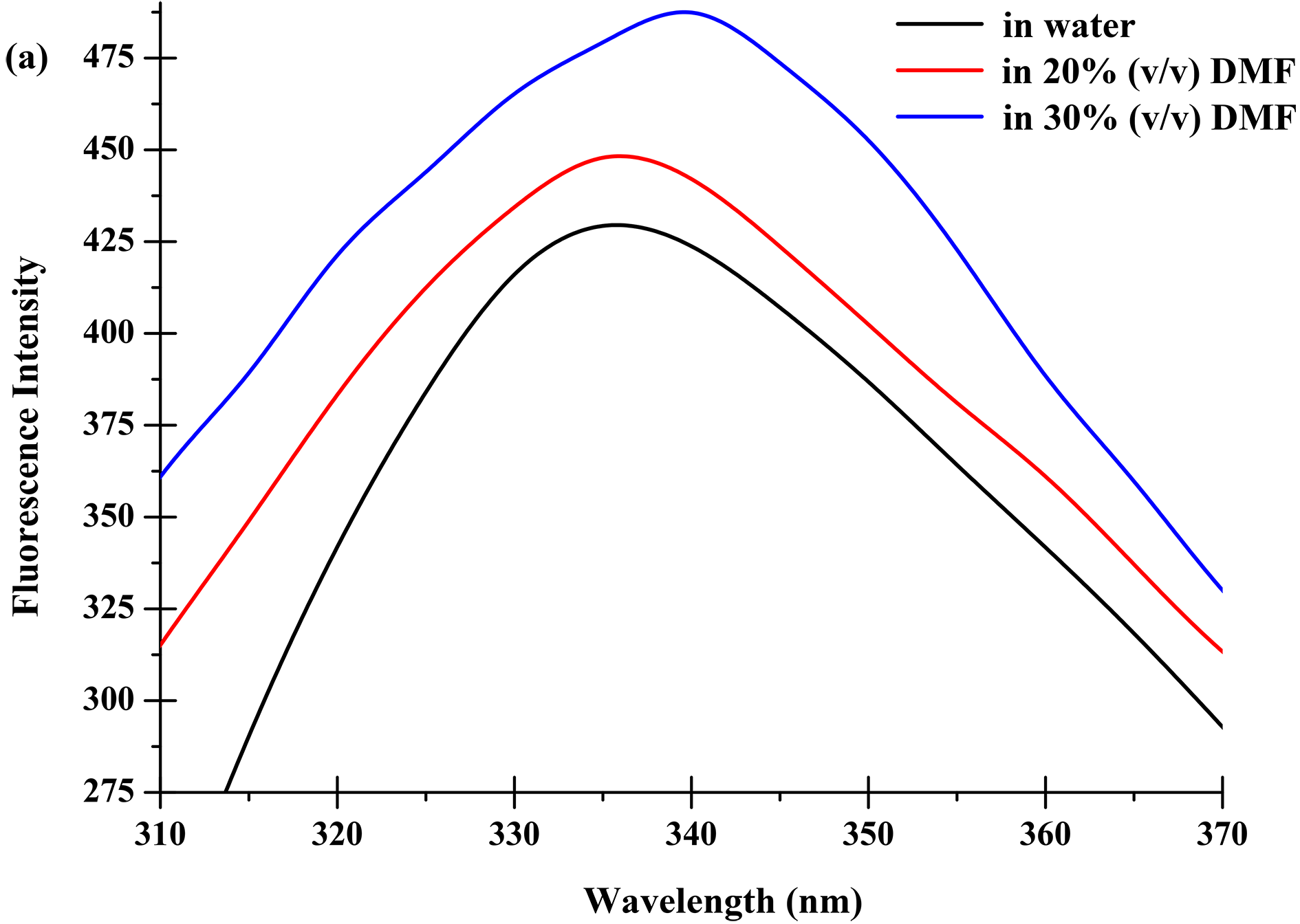

Supplement: S2 Fig — CRL was dissolved in 20% (v/v), 30% (v/v) DMF with addition of 0.5 M and 1.0 M trehalose, respectively. The fluorescence spectrum of CRL in water was as the control. The final CRL concentration was 5 μM.(c) (TIF) [file pone.0152275.s002.tif]

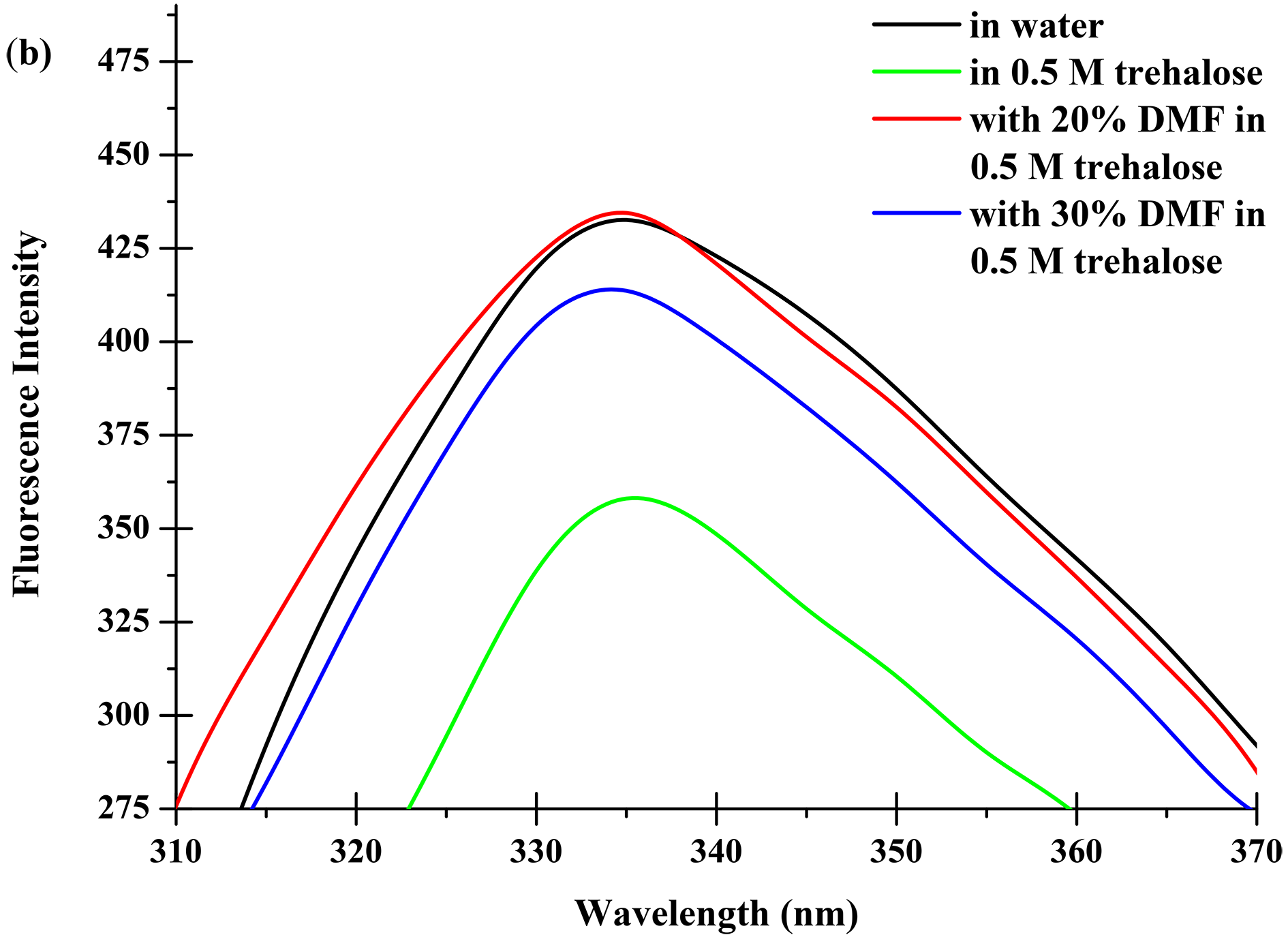

Supplement: S3 Fig — CRL was dissolved in 20% (v/v), 30% (v/v) DMF with addition of 0.5 M and 1.0 M trehalose, respectively. The fluorescence spectrum of CRL in water was as the control. The final CRL concentration was 5 μM.(c) (TIF) [file pone.0152275.s003.tif]

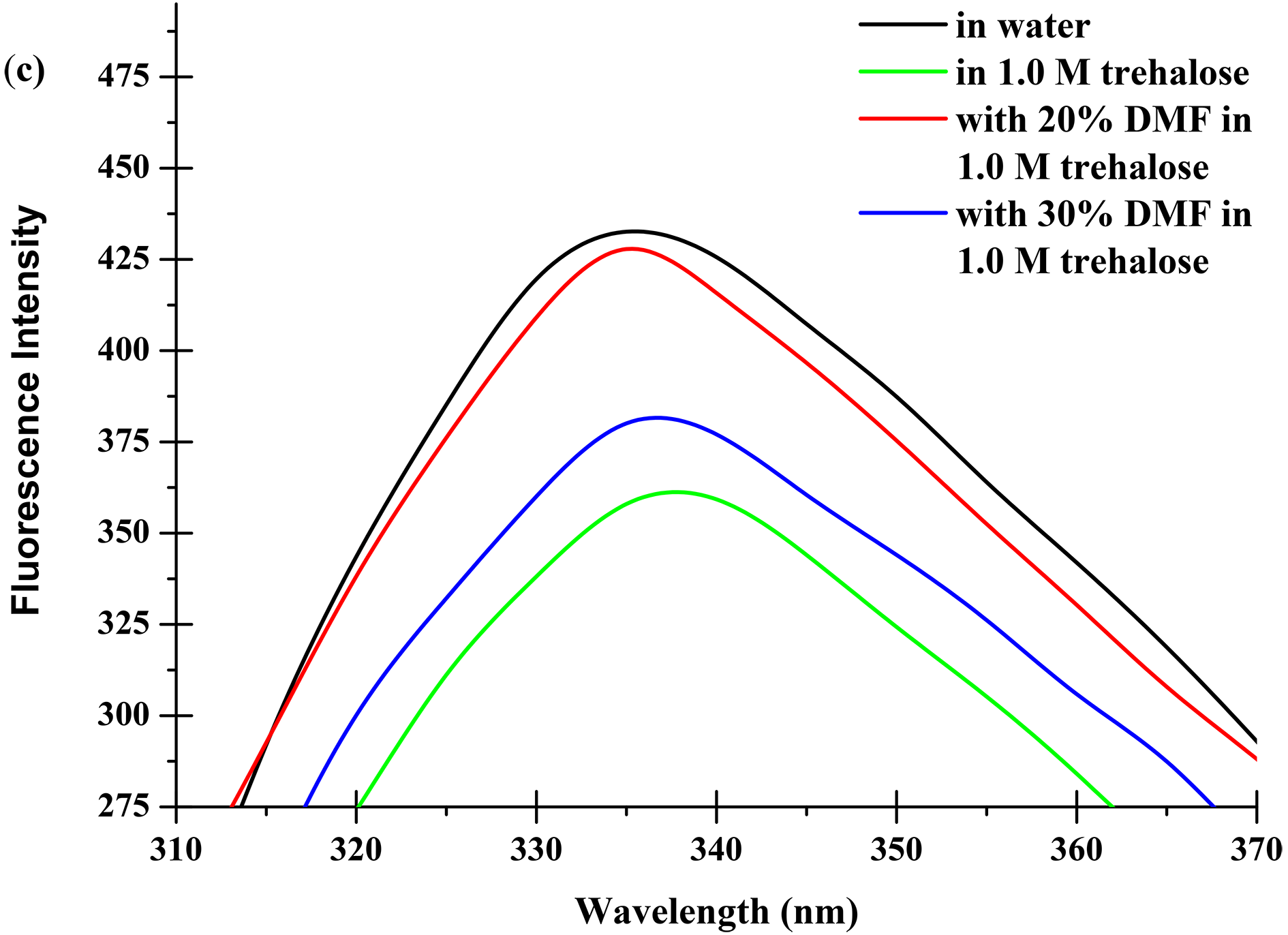

Supplement: S4 Fig — CRL was dissolved in 20% (v/v), 30% (v/v) DMF with addition of 0.5 M and 1.0 M trehalose, respectively. The fluorescence spectrum of CRL in water was as the control. The final CRL concentration was 5 μM.(c) (TIF) [file pone.0152275.s004.tif]
