# Supplementary material for: Counteraction of Trehalose on N, N-Dimethylformamide-Induced Candida rugosa Lipase Denaturation: Spectroscopic Insight and Molecular Dynamic Simulation
Source: PLoS One. 2016 Mar 31;11(3):e0152275. doi: 10.1371/journal.pone.0152275 (PMC4816565; doi:10.1371/journal.pone.0152275)
Supplement: S1 File — Table A. Summary of Simulation Systems. Table B. Energies of hydrogen bonds (Ehb) of CRL in binary and ternary solutions. (DOCX) [file pone.0152275.s005.docx]

**Table A. Summary of Simulation Systems**

| **Number of DMF molecules** | **Number of trehalose molecules** | **Number of water molecules** | **DMF concentration (mol/L)** | **Trehalose concentration (mol/L)** | **Simulation time (ns)** |
| --- | --- | --- | --- | --- | --- |
| **0** | **0** | **22189** | **0** | **0** | **12** |
| **869** | **0** | **17627** | **2** | **0** | **12** |
| **869** | **213** | **14362** | **2** | **0.5** | **12** |

**Table B. Energies of hydrogen bonds (*E*_hb_) of CRL in binary and ternary solutions.**

| **System** | ***E*_hb_**  **(Protein-DMF) ^a^** | ***E*_hb_ (Protein-Trehalose)** | ***E*_hb_ (DMF-Trehalose)** |
| --- | --- | --- | --- |
| **DMF** | **20.9** | **9.4** | **9.8** |
| **DMF+Trehalose** | **27.2** | **10.3** | **10.9** |

^a^ *E*_hb_ (kcal/mol) is calculated from 2.5×10^4^ exp(‒3.6× *d*(H‒O)), where *d*(H‒O) denotes the distance between hydrogen atom and acceptor atom. Statistical deviations are not included here since they are all smaller than 0.1 kJ/mol.
